# Characteristics of the Tongue Coating Microbiome and Its Subtype Differences in Patients with Inflammatory Bowel Disease

**DOI:** 10.3390/microorganisms14061381

**Published:** 2026-06-22

**Authors:** Jiaxin Shen, Xing Yu, Jinni Xu, Zhihua Zheng, Weiwei Zheng

**Affiliations:** 1 Endoscopic Center, The First Affiliated Hospital, Fujian Medical University, Fuzhou 350005, China; jessie@fjmu.edu.cn; 2 The First Clinical Medical College of Fujian Medical University, Fuzhou 350005, China; yuxing@fjmu.edu.cn; 3 Department of Gastroenterology, The First Affiliated Hospital, Fujian Medical University, Fuzhou 350005, China; 4 Department of Gastroenterology, National Regional Medical Center, Binhai Campus of the First Affiliated Hospital, Fujian Medical University, Fuzhou 350200, China; 5 Fujian Clinical Research Center for Liver and Intestinal Diseases, Fuzhou 350005, China; 6 Fujian Medical University, Fuzhou 350108, China; xujinni@stu.fjmu.edu.cn (J.X.); zhengzhihua@stu.fjmu.edu.cn (Z.Z.)

**Keywords:** inflammatory bowel disease, ulcerative colitis, Crohn’s disease, tongue coating, oral microbiome, 16S rRNA sequencing, oral–intestinal axis

## Abstract

Inflammatory bowel disease (IBD) is associated with microbial dysbiosis, yet subtype-specific alterations in the tongue-coating microbiome remain insufficiently characterized. In this cross-sectional study, tongue-coating samples from 158 participants (94 healthy controls [HC], 19 ulcerative colitis [UC] patients, and 45 Crohn’s disease [CD] patients) were analyzed by 16S rRNA gene amplicon sequencing. We compared alpha and beta diversity, taxonomic composition, differential taxa, exploratory random-forest feature rankings, and SPIEC-EASI co-occurrence networks. Species richness did not differ significantly among groups, whereas Shannon and Simpson indices were lower in UC than in HC and CD. Bray–Curtis and Jaccard ordinations showed significant but partially overlapping community differences among the three groups. UC was characterized by enrichment of Proteobacteria, *Neisseria*, and *Porphyromonass* (*p* < 0.001), whereas CD showed higher relative abundances of *Prevotella*, *Veillonella*, *Leptotrichia*, and TM7x. Random forest and LEfSe analyses yielded concordant candidate discriminative taxa, but no independent validation cohort was available. Network analysis suggested group-specific co-occurrence patterns, with results interpreted as statistical associations rather than direct microbial interactions. These findings support the presence of subtype-associated tongue-coating dysbiosis in IBD and identify candidate taxa for future validation.

## 1. Introduction

Inflammatory bowel disease (IBD) encompasses a diverse array of complex, chronic, and relapsing inflammatory disorders of the gastrointestinal tract, with ulcerative colitis (UC) and Crohn’s disease (CD) being the two predominant subtypes [1,2]. Recent decades have witnessed a notable increase in the global incidence of IBD, positioning it as a significant public health challenge within the domain of digestive diseases [3]. Epidemiological forecasts suggest that emerging industrialized nations will experience a transition over the next two decades, evolving from a period characterized by rising incidence and relatively low prevalence to one marked by a consistently increasing prevalence of the disease [4]. In China, the incidence of IBD has similarly shown a consistent year-on-year rise, alongside a clear trend towards a younger age of onset, primarily affecting young and middle-aged adults, thereby exacerbating the disease burden [5,6]. Moreover, due to the considerable heterogeneity of the disease and the incomplete optimization of personalized therapeutic approaches, a significant number of patients exhibit inadequate responses to current pharmacological treatments or experience secondary loss of response [7]. As a result, the identification of more robust, interpretable, and clinically relevant biomarkers, particularly in light of disease heterogeneity, has emerged as a pivotal focus in IBD research.

Although the etiology of IBD is not yet fully understood, there is a growing consensus that it arises from complex interactions among multiple distinct factors [8]. Among these factors, intestinal dysbiosis is recognized as a crucial mechanism that drives the persistence and exacerbation of mucosal inflammation [9]. The emergence of the “oral–gut axis” concept has broadened research perspectives from isolated intestinal ecosystems to encompass the continuous microbial network throughout the entire digestive tract [10]. The oral cavity, as the initial entry point of the gastrointestinal system, not only serves as a significant reservoir of microorganisms but may also act as a primary source contributing to the remodeling of microbiota associated with IBD [11]. Within the various oral microenvironments, the dorsal surface of the tongue demonstrates a relatively stable biofilm structure and favorable reproducibility for sampling; in contrast to saliva, which is a mixed biological sample, the dorsal tongue more accurately reflects a stable state of oral colonization, thereby providing a more suitable observational platform for upstream microecological disturbances [12].

Preliminary investigations, including studies on adult saliva, pre-treatment pediatric dorsal tongue samples, and oral-fecal paired analyses, have collectively indicated varying degrees of community dysbiosis at the oral site in patients with IBD, with certain alterations correlating with disease activity and showing potential diagnostic value [13]. However, several significant limitations persist in the current body of evidence. First, most studies have treated IBD as a singular entity in comparison to healthy controls, lacking a parallel analysis of UC and CD within a unified investigative framework; as a result, subtype-specific oral microecological distinctions remain inadequately characterized. Second, the term “oral microbiota” does not represent a homogeneous concept—distinct oral ecological niches exhibit considerable variability in community composition, stability, and responsiveness to disease; without explicit specification of sampling sites, the comparability and generalizability of findings are substantially limited [14,15]. Third, existing research has predominantly focused on alpha and beta diversity metrics or the screening of individual differential taxa, with insufficient integration of community composition, multi-method differential analysis, co-occurrence networks, and cross-ecological continuity evidence, thus hindering the identification of bacterial genera that are genuinely stable, reproducible, and biologically interpretable as candidate signals. Finally, while mechanistic studies have offered valuable insights into how bacteria originating from the oral cavity may translocate to and colonize the intestine under inflammatory conditions, systematic investigations that can simultaneously connect “oral-site abnormalities,” “intestinal-site indicators,” and “specific candidate taxa” at the population level remain insufficient [10,16,17].

Given the aforementioned limitations, it is essential to conduct further investigations into the oral microbiota associated with IBD, specifically utilizing tongue coating as the primary sampling site. Firstly, it is crucial to perform simultaneous comparisons of the microbiota structure of tongue coatings among healthy controls (HC), patients with UC, and patients with CD within a standardized population framework and analytical methodology. This approach will minimize biases stemming from variations in sample types, sequencing protocols, and statistical techniques employed in different studies, thus facilitating a more precise identification of subtype-specific differences. Secondly, the integration of community composition, diversity metrics, differential taxon identification, and network interaction analyses is vital for advancing interpretations from mere “compositional alterations” to a comprehensive understanding of “ecological restructuring.” This shift will enhance the robustness and biological significance of the findings. Thirdly, particular attention should be directed toward “bridging taxa” that have been consistently documented in both oral and intestinal environments—especially candidate genera such as *Veillonella*, which may play a role in ectopic colonization and the amplification of inflammation. Evaluating these taxa could elucidate their potential as continuous links between oral dysbiosis and intestinal inflammation [10,17]. Fourthly, from a clinical translational perspective, sampling from tongue coatings offers distinct advantages, including non-invasiveness, convenience, and reproducibility. Should stable microbial signatures associated with specific subtypes be identified, they could serve as a valuable population-level basis for early IBD screening, risk stratification, and subsequent mechanistic investigations [12,15,18].

Consequently, this study aimed to systematically compare diversity patterns, community composition, differential taxonomic profiles, and co-occurrence network characteristics among HC, UC, and CD cohorts within a unified tongue-coating microbiome framework. We hypothesized that UC and CD would exhibit partially distinct tongue-coating microbial signatures rather than a single undifferentiated IBD-associated pattern, and that these signatures would be most appropriately interpreted as candidate biomarkers requiring independent validation.

## 2. Materials and Methods

### 2.1. Study Population and Tongue-Coating Sample Collection

This cross-sectional study was conducted at the First Affiliated Hospital of Fujian Medical University between July 2025 and January 2026. Patients with active inflammatory bowel disease (IBD), including ulcerative colitis (UC) and Crohn’s disease (CD), were consecutively recruited according to predefined inclusion and exclusion criteria, together with age- and sex-matched healthy controls (HCs). Eligible patients were newly presenting, aged ≥ 14 years, and fulfilled established diagnostic criteria for UC or CD [19]. Disease activity was defined using disease-specific clinical indices, with a Crohn’s Disease Activity Index (CDAI) score > 150 and <450 for CD and a total Mayo score of 3–10 for UC [20,21]. Patients were excluded if they had intestinal obstruction, a high-output fistula, or had received glucocorticoids, immunosuppressants, biologics, antimicrobial agents, or probiotics within 3 months before enrollment. Healthy controls were age- and sex-matched individuals without gastrointestinal diseases or severe organ insufficiency. In total, 158 participants were included in the final analysis, comprising 94 HCs, 19 UC patients, and 45 CD patients. The group imbalance reflected the clinical availability of treatment-naive active UC and CD cases during the study period and was explicitly considered when interpreting UC-specific comparisons. The study protocol was approved by the Ethics Committee of the First Affiliated Hospital of Fujian Medical University [Approval No. MRCTA, ECFAH of FMU (2022) 317], and written informed consent was obtained from all participants prior to enrollment.

Baseline demographic matching was performed for age and sex. Body mass index and smoking status were reviewed where available, but detailed dietary intake, oral hygiene behavior, and periodontal indices were not uniformly collected for all participants and therefore could not be included as covariates in the primary analyses. This limitation is acknowledged in our study, and the present results should be interpreted as microbiome associations observed within a cross-sectional cohort rather than as confounder-adjusted causal effects.

Tongue-coating samples were collected from all participants using sterile swabs according to a standardized protocol. Participants were instructed to rinse their mouths with normal saline 30 min before sampling. The middle dorsum of the tongue with visible coating was swabbed while avoiding contact with other oral sites to minimize contamination. Samples were immediately snap-frozen in liquid nitrogen and stored at −80 °C until further processing.

### 2.2. DNA Extraction and 16S rRNA Sequencing

Total genomic DNA was extracted from tongue-coating samples using a modified sodium dodecyl sulfate (SDS)-based method. Briefly, swab heads were immersed in SDS-containing lysis buffer and incubated at 65 °C for 1 h to disrupt microbial cells. DNA was purified by phenol-chloroform-isoamyl alcohol (25:24:1, *v*/*v*) extraction, precipitated with isopropanol, washed with 70% ethanol, air-dried, and resuspended in sterile water. DNA concentration and purity were assessed before amplification, and DNA was diluted to 1 ng/μL with sterile water. The V3–V4 region of the bacterial 16S rRNA gene was amplified using barcoded 341F/806R primers (341F: 5′-CCTAYGGGRBGCASCAG-3′; 806R: 5′-GGACTACNNGGGTATCTAAT-3′). PCR amplification was performed in a 30 μL reaction volume containing 15 μL Phusion High-Fidelity PCR Master Mix with GC Buffer (New England Biolabs, Ipswich, MA, USA), 0.2 μM of each primer, and approximately 10 ng of template DNA. The thermal cycling conditions were as follows: initial denaturation at 98 °C for 1 min; 30 cycles of denaturation at 98 °C for 10 s, annealing at 50 °C for 30 s, and extension at 72 °C for 60 s; followed by a final extension at 72 °C for 5 min. PCR products were examined on 2% agarose gels, pooled at equidensity ratios, and purified using the GeneJET Gel Extraction Kit (Qiagen, Hilden, Germany). Sequencing libraries were generated using the TruSeq^®^ DNA PCR-Free Sample Preparation Kit (Illumina, San Diego, CA, USA), and library quality was assessed using a Qubit 3.0 Fluorometer (Invitrogen, Carlsbad, CA, USA) and an Agilent Bioanalyzer 5400 system (Agilent Technologies, Santa Clara, CA, USA). Libraries were sequenced on an NovaSeq 6000 platform (Illumina, San Diego, CA, USA) using paired-end PE250 mode (2 × 250 bp).

Raw demultiplexed paired-end reads were quality-filtered and denoised using the QIIME 2/DADA2 workflow to generate amplicon sequence variants (ASVs) [22,23]. Chimeric sequences and low-quality reads were removed during denoising, and the resulting ASV table was used for downstream analyses. Taxonomic assignment was performed using the QIIME 2 feature-classifier classify-sklearn method with a trained naive Bayes classifier and the default confidence threshold of 0.7. The classifier was constructed from the SILVA 138 release with 99% full-length reference sequences and taxonomy files downloaded from the QIIME 2 2024.10 data resources (https://docs.qiime2.org/2024.10/data-resources/, accessed on 1 October 2025). To match the amplified region, reference reads were first extracted using the 341F primer (5′-CCTAYGGGRBGCASCAG-3′) and 806R primer (5′-GGACTACNNGGGTATCTAAT-3′) with read orientation set to both, and a region-specific classifier was then trained using feature-classifier fit-classifier-naive-bayes. The bacterial taxonomic classifier used for ASV annotation was silva-138-99-341-806-nb-classifier.qza. The ASV abundance table, sample metadata, and taxonomic annotation tables were imported into R for downstream analyses. Taxonomic labels were cleaned and standardized using the tidy_taxonomy function in the microeco package, and a microtable object was constructed to integrate abundance profiles, taxonomy, and sample grouping information [24].

### 2.3. Microbiome and Statistical Analyses

All microbiome analyses, statistical tests, and visualizations were performed in R (version 4.5.2). Alpha diversity was assessed using the phyloseq and vegan packages, with Chao1 and Observed ASVs used to evaluate richness and Shannon and Simpson indices used to assess community diversity and evenness [25]. Group-wise differences among HC, UC, and CD were evaluated using the Kruskal–Wallis rank-sum test, followed by pairwise Wilcoxon rank-sum tests, and were visualized using raincloud plots. For beta-diversity analysis, ASV abundances were transformed into relative abundances prior to calculation of Bray–Curtis and Jaccard distance matrices. Principal coordinates analysis (PCoA) was performed to visualize between-group differences, with convex hulls and marginal density plots used to illustrate group distributions. Group differences in community structure were tested by permutational multivariate analysis of variance (PERMANOVA; adonis2, 999 permutations), and homogeneity of multivariate dispersion was assessed using betadisper. Test statistics, PERMANOVA R2 values, and adjusted *p* values were considered together when interpreting statistical evidence.

Microbial community composition was summarized at the phylum and genus levels using stacked bar plots, heatmaps of the top 30 genera, and Venn diagrams of shared and unique ASVs among the three groups. Differential and discriminative microbial features were evaluated mainly at the genus level, with species-level results treated as exploratory because short-read 16S rRNA amplicon sequencing has limited resolution for definitive species assignment. LEfSe analysis was conducted using microeco::trans_diff(method = ‘lefse’), with group as the class variable, a significance threshold of alpha = 0.05, and an LDA score threshold of 2.0. Random forest analysis was used as an exploratory feature-ranking approach rather than as a clinically validated diagnostic classifier; feature importance was ranked according to MeanDecreaseGini and interpreted alongside LEfSe and abundance patterns. No independent external validation cohort was available. To investigate group-specific ecological associations, microbial co-occurrence networks were constructed separately for the HC, UC, and CD groups. Taxa present in fewer than 10% of samples within each group or with a total abundance < 10 were removed, and the top 200 taxa ranked by total abundance were retained for network inference. Networks were inferred using the SPIEC-EASI framework with the Meinshausen–Buhlmann neighborhood selection method, and the optimal sparsity parameter was selected using the Stability Approach to Regularization Selection (StARS), with 50 repetitions and a stability threshold of 0.05. Network topological properties, including node degree, were calculated using the igraph package, and networks were visualized using the ggraph package. Because SPIEC-EASI estimates conditional associations from compositional data, network edges were interpreted as putative co-occurrence relationships and not as evidence of direct interaction or causality. Unless otherwise specified, all statistical tests were two-sided, and *p* values from pairwise comparisons were adjusted using the Benjamini–Hochberg method. A corrected *p* value <0.05 was considered statistically significant.

### 2.4. Sequencing Data Quality Control

To ensure the reliability of sequencing data, quality control (QC) was performed for all samples. Sequencing was carried out on the Illumina MiSeq platform using paired-end PE250 mode (2 × 250 bp). Raw FASTQ files were evaluated to determine per-sample sequencing depth, read length distribution, GC content, N content, Q20, and Q30 values. After quality filtering, chimera removal, and denoising, ASV feature tables were generated for subsequent analysis. Because this study applied targeted bacterial 16S rRNA gene amplicon sequencing rather than shotgun metagenomic sequencing, alignment to the human genome was not performed. Instead, the proportion of sequences assigned to bacteria was summarized using taxonomic annotation results. Detailed sequencing QC statistics, including per-sample read counts, read length, base quality, and bacterial assignment rates, are provided in Supplementary Methods and Supplementary Table S1.

## 3. Results

### 3.1. Alpha Diversity Analysis

**Figure 1:**
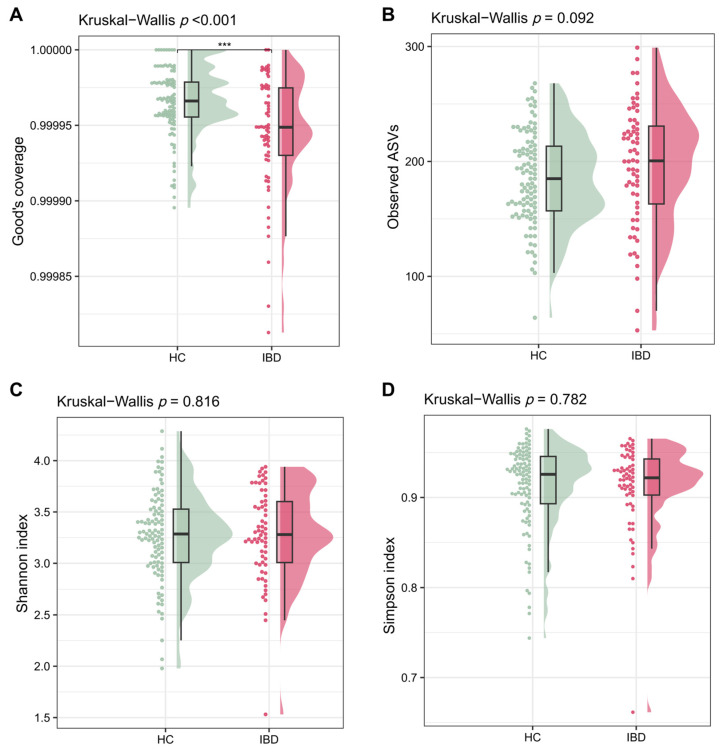
Comparison of alpha diversity indices among the three cohorts. Caption: Distribution of alpha diversity metrics across HC, UC, and CD groups. (**A**): Chao1; (**B**): Observed ASVs; (**C**): Shannon; (**D**): Simpson. Each panel superimposes individual data points, box plots (median and interquartile range), and density distributions. Overall intergroup differences were assessed using Kruskal–Wallis tests; pairwise comparisons were performed using Wilcoxon rank-sum tests with Benjamini–Hochberg correction. Horizontal lines and asterisks denote adjusted pairwise significance levels where applicable. *** *p* < 0.001, where shown.

Comparative analyses of alpha diversity indices among the three cohorts—healthy controls (HC), ulcerative colitis (UC), and Crohn’s disease (CD)—were depicted through raincloud plots. No statistically significant differences were identified among the groups regarding species richness metrics, such as the Chao1 index and observed ASVs (Figure 1A,B). Conversely, significant intergroup differences were noted in community evenness and diversity metrics, specifically the Shannon index (*p* = 0.010) and the Simpson index (*p* = 0.006) (Figure 1C,D). Pairwise comparisons indicated that the UC group exhibited lower Shannon and Simpson indices compared with both the HC and CD groups, suggesting a reduction in community evenness among UC patients. The CD group showed diversity values closer to those of HCs for these metrics.

### 3.2. Beta Diversity Analysis

**Figure 2:**
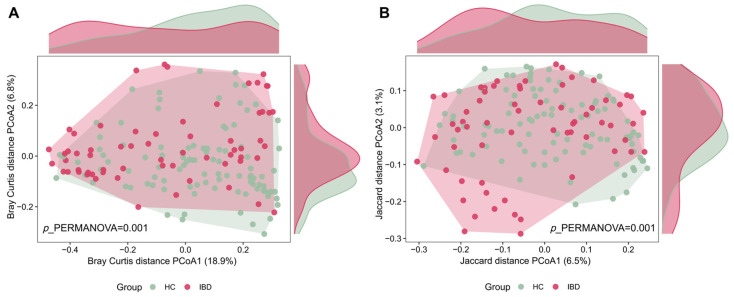
Principal coordinate analysis of beta diversity and intergroup difference testing across the three cohorts. Caption: PCoA results based on (**A**) Bray–Curtis distance and (**B**) Jaccard distance. Individual points represent samples, color-coded by group (HC, UC, CD); semi-transparent polygons denote group-specific convex hull boundaries; marginal density distributions are displayed along the top and right axes. Percentage variation explained is indicated in parentheses on axis labels. PERMANOVA was performed with 999 permutations, and group dispersion was assessed using betadisper.

Principal coordinate analysis (PCoA) showed statistically significant differences in community composition among the three cohorts using both Bray–Curtis and Jaccard distance metrics (PERMANOVA *p* = 0.001 for both; Figure 2A,B). The marginal density distributions and convex hull boundaries indicated group-level shifts with partial overlap among individual samples, supporting subtype-associated differences while also demonstrating substantial within-group heterogeneity.

### 3.3. Microbial Composition Analysis

**Figure 3:**
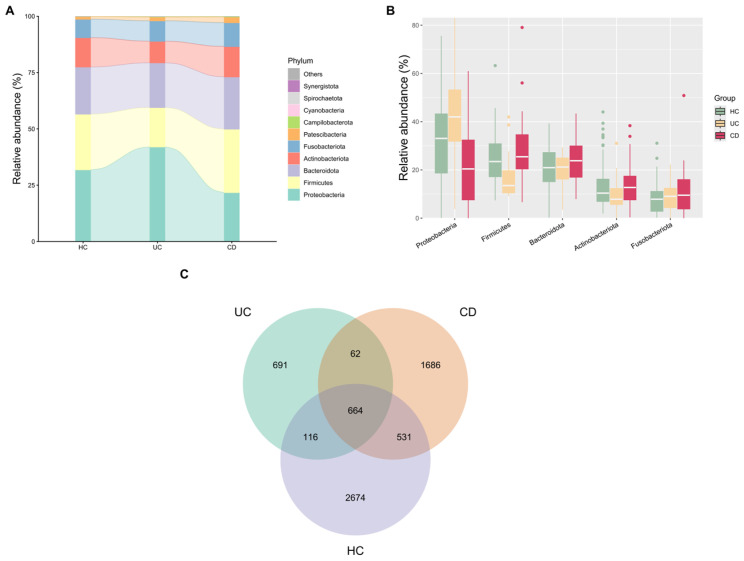
Phylum-level community composition and abundance distribution of major phyla. Caption: (**A**): Stacked bar plot showing relative abundances at the phylum level across the three cohorts (major phyla and “Others” category displayed). (**B**): Box plots of relative abundance (%) for predominant phyla (Proteobacteria, Firmicutes, Bacteroidota, Actinobacteriota, Fusobacteriota) across HC, UC, and CD groups, illustrating intergroup distributional differences. (**C**): Venn diagram illustrating the distribution and overlap of differentially abundant taxa (or ASVs/OTUs) among the HC, UC, and CD groups.

At the phylum level (Figure 3), pronounced between-group differences were observed in the composition of dominant bacterial phyla across the three cohorts (Figure 3A). Boxplot analyses of the major phyla (Figure 3B) indicated that the UC group exhibited an overall higher relative abundance of Proteobacteria, whereas Firmicutes were depleted in UC but enriched in CD. Moreover, Bacteroidota, Actinobacteriota, and Fusobacteriota displayed distinct distributional patterns among the groups (Figure 3B).

**Figure 4:**
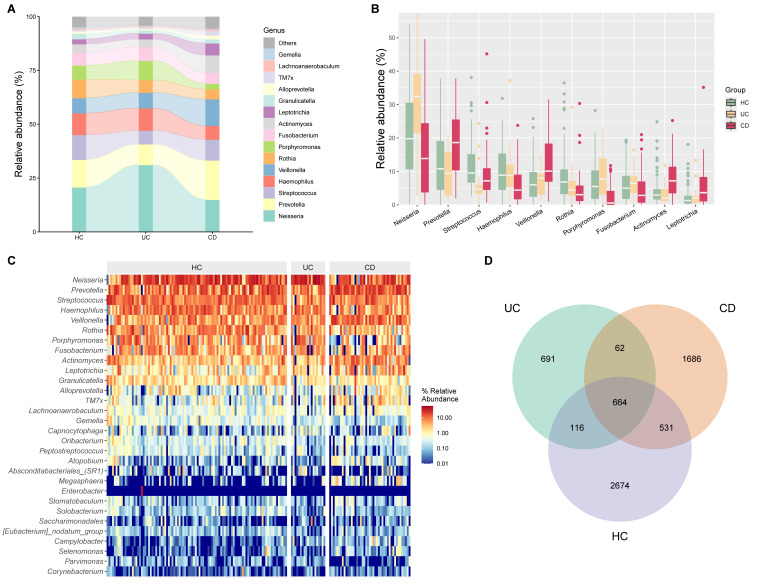
Genus-level community structure, abundance differences in representative taxa, and shared ASVs. Caption: (**A**): Stacked bar plot of relative abundances at the genus level; (**B**): Box plots of relative abundance (%) for representative predominant genera (e.g., *Neisseria*, *Prevotella*, *Streptococcus*, *Haemophilus*, *Veillonella*) across the three cohorts; (**C**): Heatmap of genus-level relative abundances (group-stratified display, color intensity on logarithmic scale); (**D**): Venn diagram illustrating shared and group-specific ASVs among HC, UC, and CD groups.

At the genus level (Figure 4), differences in the composition of dominant bacterial genera were observed among the three cohorts (Figure 4A–C). Boxplot comparisons showed increased abundances of *Neisseria* and *Porphyromonas* in the UC group, whereas the CD group displayed higher relative abundances of *Prevotella*, *Veillonella*, and *Leptotrichia*. In contrast, the HC group was characterized by higher levels of *Streptococcus*, *Haemophilus*, and *Rothia* (Figure 4B). These cohort-associated patterns were further supported by the sample-level heatmap, which demonstrated group-related clustering with overlap among samples (Figure 4C). The Venn diagram (Figure 4D) revealed 664 ASVs shared by all three groups, along with 2674, 691, and 1686 unique ASVs in HC, UC, and CD, respectively. Collectively, HC exhibited the largest number of group-specific features, whereas UC showed the fewest, indicating intergroup differences in characteristic community composition and in the distribution of core versus cohort-associated taxa.

### 3.4. Differential Taxa and Discriminative Feature Analysis

**Figure 5:**
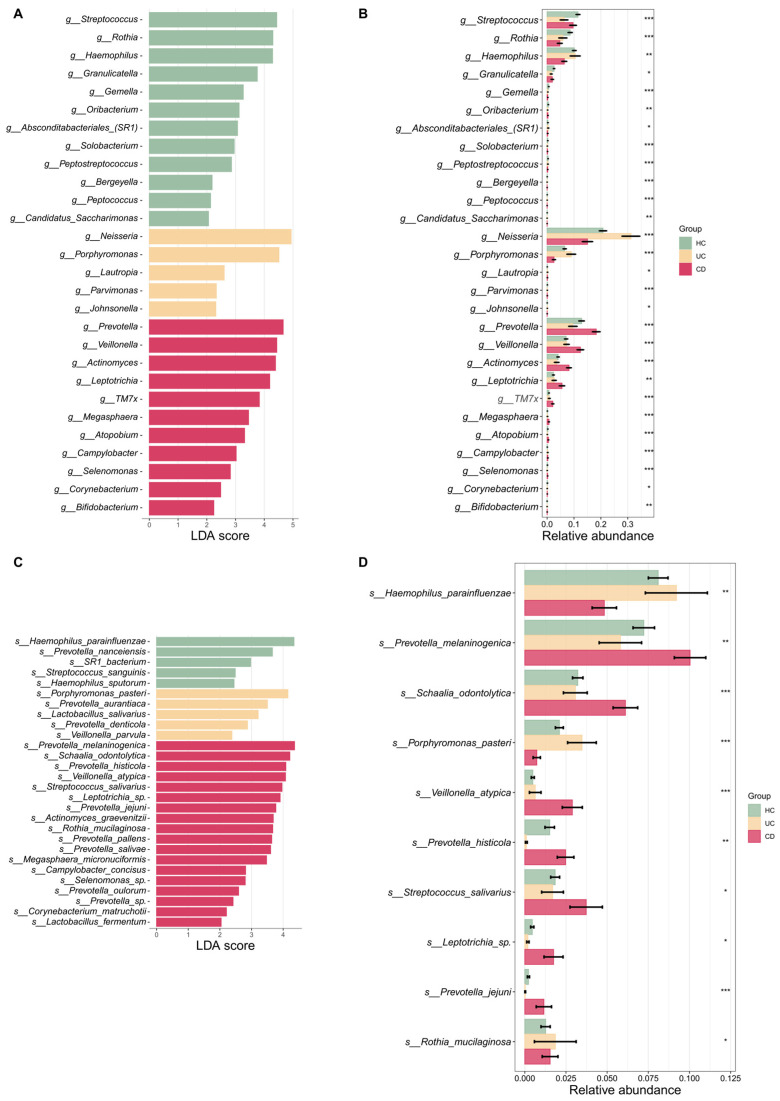
Differential genera and species identified by LEfSe analysis. Caption: (**A**): LDA score bar plot at the genus level using an LDA threshold of 2.0; (**B**): Relative abundance comparisons (mean ± SE) of corresponding differential genera with significance annotations; (**C**): LDA score bar plot at the species level using an LDA threshold of 2.0; (**D**): Relative abundance comparisons of corresponding differential species. Species-level results are exploratory because 16S rRNA amplicon sequencing has limited species-level resolution. * *p* < 0.05, ** *p* < 0.01, *** *p* < 0.001 where shown.

LEfSe analysis at the genus level (LDA score threshold = 2.0; Figure 5A,B) identified taxa enriched across groups. The HC group was predominated by *Streptococcus*, *Rothia*, *Haemophilus*, *Granulicatella*, and *Gemella*. The UC group showed enrichment of *Neisseria*, *Porphyromonas*, *Lautropia*, *Parvimonas*, and *Johnsonella*. In contrast, the CD group exhibited enrichment of *Prevotella*, *Veillonella*, *Actinomyces*, *Leptotrichia*, TM7x, *Megasphaera*, *Atopobium*, *Campylobacter*, *Selenomonas*, *Corynebacterium*, and *Bifidobacterium* (Figure 5A). Relative abundance comparisons further demonstrated statistically significant intergroup differences across multiple taxonomic units (Figure 5B).

At the species level (Figure 5C,D), results were considered exploratory because 16S rRNA amplicon sequencing cannot reliably resolve all bacterial species. Within this limitation, the HC group was enriched for *Haemophilus parainfluenzae* and *Streptococcus sanguinis*, whereas the UC group was enriched for *Porphyromonas pasteri*, *Prevotella aurantiaca*, and *Prevotella denticola*. The CD group showed enrichment of *Veillonella atypica*, *Schaalia odontolytica*, *Actinomyces graevenitzii*, *Leptotrichia* sp., and multiple *Prevotella* species, including *P. melaninogenica*, *P. salivae*, *P. pallens*, *P. histicola*, *P. jejuni*, and *P. oulorum* (Figure 5C,D).

**Figure 6:**
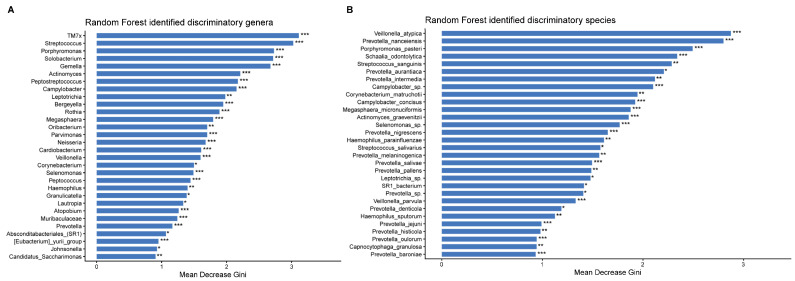
Candidate discriminative genera and species identified by random forest feature ranking. Caption: (**A**): Genus-level features ranked by MeanDecreaseGini; (**B**): Species-level exploratory features ranked by MeanDecreaseGini. Random forest results are interpreted as exploratory feature importance rankings rather than as externally validated diagnostic performance. * *p* < 0.05, ** *p* < 0.01, *** *p* < 0.001 where shown.

**Figure 7:**
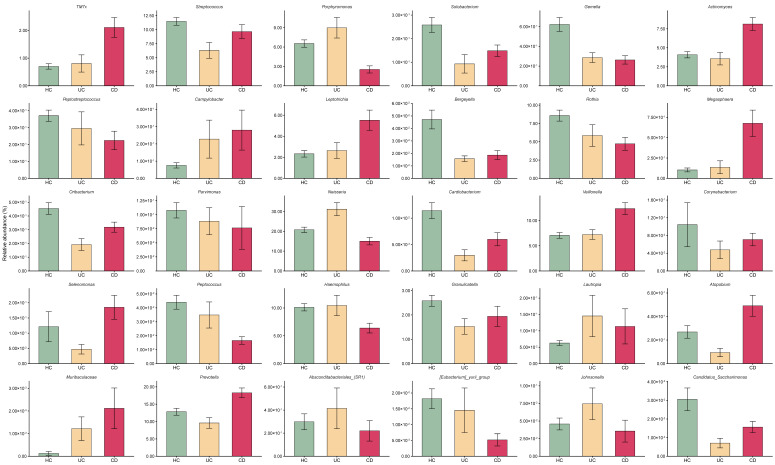
Abundance distributions of candidate discriminative genera selected by random forest feature ranking across the three cohorts. Caption: Mean relative abundances (%) with error bars for the top-ranked genera, displayed individually across HC, UC, and CD groups. The figure is intended to show abundance directionality for exploratory features.

**Figure 8:**
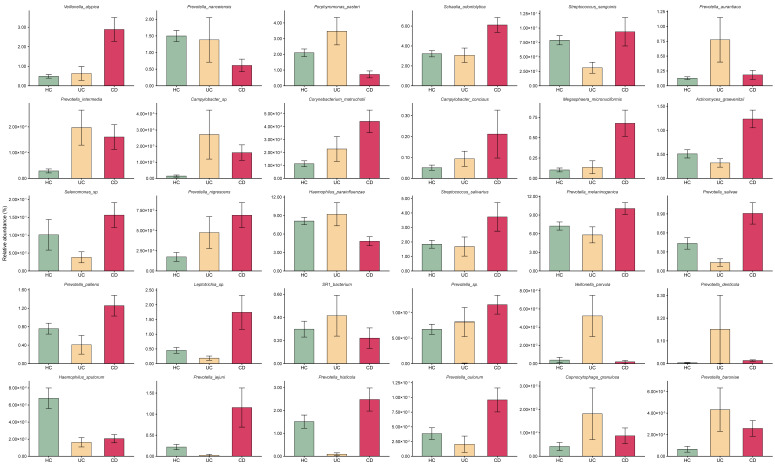
Abundance distributions of candidate discriminative species selected by random forest feature ranking across the three cohorts. Caption: Mean relative abundances (%) with error bars for the top-ranked species, displayed individually across HC, UC, and CD groups. Species-level assignments from 16S rRNA amplicon data should be interpreted cautiously.

Random forest feature ranking at the genus level prioritized TM7x, *Streptococcus*, *Porphyromonas*, *Solobacterium*, *Gemella*, and *Actinomyces* as informative candidate features (high MeanDecreaseGini; Figure 6A), with their abundance distributions shown across groups in Figure 7. At the species level, *Veillonella atypica*, *Prevotella nanceiensis*, *Porphyromonas pasteri*, *Schaalia odontolytica*, and *Streptococcus sanguinis* were identified as high-importance exploratory features (Figure 6B), and their group-wise abundance patterns are depicted in Figure 8. Overall, LEfSe and random forest analyses were concordant in suggesting group-associated candidate taxa: HC was characterized by enrichment of *Streptococcus*, *Haemophilus*, and *Rothia*; UC by *Neisseria* and *Porphyromonas*; and CD by *Prevotella*, *Veillonella*, *Actinomyces*, *Leptotrichia*, and TM7x (Figure 5, Figure 6, Figure 7 and Figure 8). These findings should be interpreted as feature-ranking results requiring external validation, not as validated diagnostic biomarkers.

### 3.5. Microbial Co-Occurrence Networks

**Figure 9:**
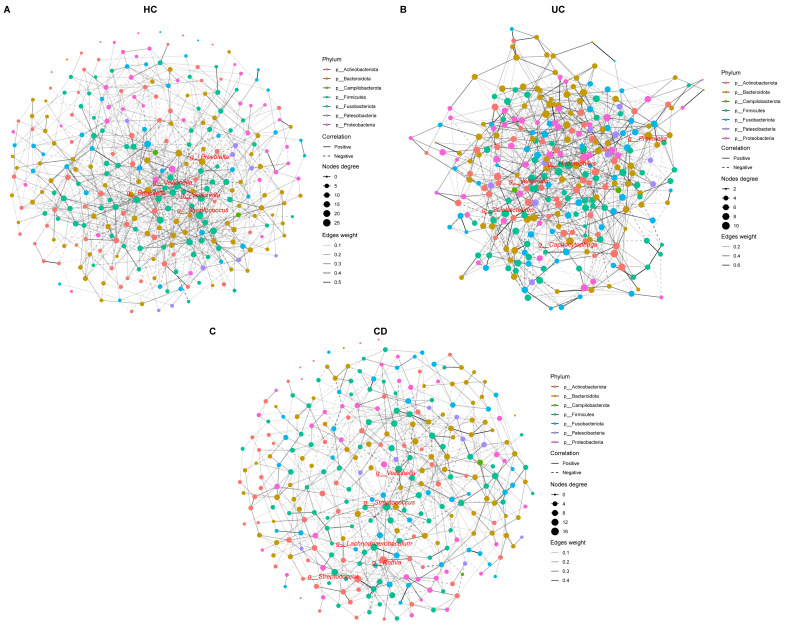
Comparison of microbial co-occurrence networks across the three cohorts. Caption: Co-occurrence networks for (**A**): HC group; (**B**): UC group; and (**C**): CD group. Node colors indicate phylum affiliation; node size represents degree (number of connections); edges represent SPIEC-EASI-inferred conditional associations, with solid lines denoting positive associations and dashed lines denoting negative associations. Network hubs are high-degree taxa within inferred association networks and should not be interpreted as confirmed causal driver species.

Group-specific microbial co-occurrence networks were subsequently constructed (nodes colored by phylum; node size proportional to degree; edges indicating positive and negative conditional associations; Figure 9A–C). The HC network displayed a wider distribution of node degrees and increased edge density, consistent with a more complex inferred association structure (Figure 9A). The UC network exhibited a narrower degree range (maximum approximately 10) and a more centralized topology, with high-degree taxa including *Prevotella*, *Haemophilus*, *Veillonella*, *Solobacterium*, and *Capnocytophaga* (Figure 9B). The CD network showed an intermediate degree range (maximum approximately 16) relative to HC and UC, with *Veillonella*, *Streptococcus*, *Rothia*, and *Lachnoanaerobaculum* emerging as major hub taxa (Figure 9C). Collectively, these results indicate that the three cohorts differ not only in microbial composition but also in inferred co-occurrence architecture. However, these network patterns represent statistical associations and should not be interpreted as direct microbial interactions or causal ecological relationships.

## 4. Discussion

IBD is a complex, multifactorial disorder. Conventional IBD research has largely centered on the intestinal microbiota, and ecological dysbiosis within gut microbial communities is widely recognized as an important correlate of mucosal inflammation. More recently, growing attention has been directed toward the oral–gut axis, which proposes that the oral microbiota may act as a reservoir of organisms capable of reaching the intestine and interacting with intestinal microecology [16,26]. In this cross-sectional study, tongue-coating profiles differed among HC, UC, and CD cohorts, suggesting subtype-associated oral microbial alterations. Because of the observational design, these findings should be interpreted as associations rather than evidence that oral dysbiosis causes intestinal inflammation.

Ecological stability of the oral microbiome is reflected in the balance between microbial diversity and community structure. In the present analysis, alpha diversity indicated that the UC cohort had the lowest Shannon and Simpson indices among the three groups, whereas the CD cohort exhibited indices closer to those of HCs. In parallel, beta-diversity analyses based on Bray–Curtis and Jaccard distances demonstrated statistically significant differences among all three groups. These community-level findings suggest restructuring of the tongue-coating microbiota in IBD, while the observed overlap among individuals also indicates heterogeneity within each clinical category. Consistent with these findings, prior studies have reported that oral microbial communities in IBD differ from those of healthy individuals, often with reduced alpha diversity [18,27]. Such community-level imbalance may be linked to local oral inflammation, systemic inflammatory states, medication exposure, or unmeasured lifestyle and oral-health factors.

At the phylum level, compositional profiling revealed an increased relative abundance of Proteobacteria accompanied by a reduction in Firmicutes in the UC group. Many Proteobacteria are facultative anaerobes capable of using nitrate and related compounds as alternative electron acceptors in inflamed environments; their expansion has been associated with oxidative and inflammatory stress [28]. In UC, systemic inflammation may therefore be associated with ecological conditions that favor Proteobacteria, although this study cannot determine directionality. Notably, Firmicutes displayed divergent behavior across IBD subtypes—decreasing in UC but increasing in CD—suggesting that distinct disease entities may be associated with different oral microbial states. These phylum-level observations should be considered descriptive patterns rather than independently validated biomarkers.

At the genus level, UC was characterized by enrichment of *Neisseria* and *Porphyromonas*, whereas CD exhibited higher abundances of *Prevotella*, *Veillonella*, and *Leptotrichia*, broadly aligning with prior reports [29]. *Neisseria* species are common commensals of the upper respiratory tract and oral cavity [30], and their increased abundance in UC may indicate adaptive expansion under inflammatory pressure. In addition, Proteobacteria-associated taxa, including established periodontal pathogens such as *Fusobacterium nucleatum*, are notable for producing virulence factors that can compromise epithelial barriers. These organisms are frequently linked to oral inflammation and multiple systemic conditions and may influence systemic physiology through transient bacteremia [31]. *Prevotella* and *Veillonella* are also repeatedly reported as enriched in oral microbiome studies of IBD; they may contribute to metabolic remodeling within inflammatory microenvironments, and their overrepresentation has been associated with inflammatory activity in IBD. Together, these findings underscore close interconnections between oral and intestinal ecosystems. Importantly, in contrast to our results, a pediatric IBD study reported reduced abundances of *Prevotella*, Fusobacterium, *Leptotrichia*, and *Porphyromonas* in IBD patients, with CD samples diverging more strongly from healthy controls than UC samples [12]. These discrepancies may reflect physiological and immunological differences between pediatric and adult populations. Relative to children—who may be treatment-naïve or have shorter exposure durations—adult cohorts may represent microbiota that have chronically adapted to prolonged systemic inflammation and medication histories. Moreover, differences in immune maturation may lead to age-dependent oral immune responses and varying degrees of commensal clearance in the context of IBD.

The identification of reproducible candidate markers is pivotal for translating microbiome research into clinical practice. In this study, the HC cohort was characterized by enrichment of *Streptococcus*, *Haemophilus*, and *Rothia*; the UC cohort showed increased abundance of *Neisseria* and *Porphyromonas*; and the CD cohort exhibited enrichment of *Prevotella*, *Veillonella*, *Actinomyces*, *Leptotrichia*, and TM7x. *Streptococcus*, *Haemophilus*, and *Rothia* constitute common oral commensals and contribute to maintenance of the oral nitrate-nitrite-nitric oxide pathway; their predominance in the HC group is consistent with a homeostatic oral microecological state [32]. LEfSe and random forest analyses yielded concordant candidate features, but these taxa should be considered preliminary and require validation in independent cohorts before clinical application. Among the candidate features, TM7x emerged as an important taxon in the CD group. As a representative member of the Candidate Phyla Radiation (CPR), TM7x is an obligate epibiont associated with *Actinomyces*. The co-enrichment of TM7x and *Actinomyces* in tongue-coating samples from CD patients suggests a possible symbiotic configuration within the CD-associated oral microenvironment; however, mechanistic implications remain speculative without functional experiments. Recent evidence indicates that TM7x can modulate host pathogenicity and immune recognition, but whether similar mechanisms operate in IBD requires further study [33]. Our findings should therefore be regarded as preliminary, associative, and hypothesis-generating rather than functionally definitive.

Microorganisms assemble dynamic ecological networks through multifaceted interspecies associations. Co-occurrence network analysis in the present work suggested that, beyond compositional shifts, inferred microbial association patterns differed among the three cohorts. The healthy oral microbial network exhibited a broader range of node degrees and greater network density, whereas the UC network was simplified and displayed a constrained degree range. The CD network showed intermediate topological features between HC and UC. These observations may reflect differences in community organization, but SPIEC-EASI networks are inferred from compositional abundance data and therefore cannot establish direct interactions, ecological stability, or causal roles for hub taxa. Accordingly, network results were used to generate hypotheses for future experimental and longitudinal studies rather than to define mechanistic drivers.

The selection of tongue coating as the sampling site carries clear methodological significance. Among oral microhabitats, the dorsal tongue surface supports a relatively stable biofilm structure, and its specialized anatomy provides an advantageous niche for diverse anaerobic and facultative anaerobic organisms. Accordingly, this site can capture oral colonization characteristics with high fidelity while offering practical advantages, including non-invasiveness, ease of sampling, and reproducibility.

Despite offering systematic insight into IBD-associated oral ecological dysbiosis, this study has several limitations. First, the cross-sectional design limits inference regarding temporal dynamics and causality; IBD follows a chronic relapsing-remitting course, during which microbial communities may evolve with disease activity and therapeutic exposure. Second, the cohorts were imbalanced, particularly for UC (*n* = 19), which may reduce power for UC-specific pairwise comparisons and increase uncertainty in feature-ranking analyses. Third, although age and sex matching were performed, detailed dietary intake, oral hygiene behavior, smoking exposure, body mass index, and periodontal status were not uniformly available for covariate-adjusted analyses. Fourth, 16S rRNA V3-V4 amplicon sequencing provides limited taxonomic resolution at the species and strain levels, and species-level assignments should therefore be interpreted cautiously. Fifth, random forest and SPIEC-EASI analyses were exploratory: random forest features were not externally validated as diagnostic biomarkers, and network edges indicate statistical associations rather than direct interactions. Lastly, because this study included only active IBD cases and individual continuous disease-activity scores were not incorporated into the final microbiome analysis dataset, we were unable to assess correlations between oral microbial features and CDAI or total Mayo scores. Future work should incorporate larger and more balanced multicenter cohorts, external validation, longitudinal disease-activity measurements, comprehensive oral-health and lifestyle covariates, and multi-omics strategies such as metagenomics and metabolomics.

## 5. Conclusions

This study suggests that patients with inflammatory bowel disease (IBD) exhibit subtype-associated alterations in the tongue-coating microbiome. The ulcerative colitis (UC) cohort showed reduced Shannon and Simpson diversity indices relative to both healthy controls (HC) and Crohn’s disease (CD), whereas beta-diversity analyses revealed significant but partially overlapping community-level differences among all three groups. Taxonomically, UC was characterized by an increased relative abundance of Proteobacteria, a depletion of Firmicutes, and enrichment of *Neisseria* and *Porphyromonas*. In contrast, CD was associated with enrichment of *Prevotella*, *Veillonella*, *Leptotrichia*, and TM7x. These candidate discriminative taxa were supported by both LEfSe and exploratory random forest feature ranking. Co-occurrence network analysis further suggested a densely interconnected topology in HCs, a simplified network architecture in UC, and an intermediate level of complexity in CD. Collectively, tongue-coating microbial features may provide non-invasive candidates for future IBD subtype-discrimination studies, but external validation and longitudinal mechanistic work are required before clinical translation.

## Data Availability

The original contributions presented in this study are included in the article/Supplementary Material. Further inquiries can be directed to the corresponding author.

## Supplementary Materials

The following supporting information can be downloaded at: https://www.mdpi.com/article/10.3390/microorganisms14061381/s1, Supplementary Figure S1. FASTQ and 16S assignment overview across HC, UC, and CD groups. Panels show raw read pairs, raw Q20, raw Q30, and the percentage of ASV feature-table reads assigned to Bacteria. Supplementary Table S1A. Group-level summary. Supplementary Table S1B. Field legend. Supplementary Table S1C. Per-sample core sequencing and QC metrics. Supplementary Table S1D. Raw sequencing-report samples excluded from the manuscript analysis.
