# Supplementary material for: Characteristics of the Tongue Coating Microbiome and Its Subtype Differences in Patients with Inflammatory Bowel Disease
Source: Microorganisms. 2026 Jun 22;14(6):1381. doi: 10.3390/microorganisms14061381 (PMC13304174; doi:10.3390/microorganisms14061381)
Supplement: Supplementary file 1 [file microorganisms-14-01381-s001.zip › microorganisms-4296189-supplementary.pdf]

Supplementary Material: Sequencing Depth, Read Length, FASTQ QC, and 16S Taxonomic Assignment

This supplementary material summarizes the newly added sequencing and quality-control data for the HC/UC/CD manuscript cohort (164 samples: HC=100, UC=19, CD=45). The full spreadsheet version is also provided as Supplementary_Table_S1_sequencing_QC_mapping.xlsx.

Supplementary Methods

Sequencing was performed using Illumina paired-end PE250 mode (2 x 250 bp). Raw paired-end FASTQ files were assessed per sample to summarize sequencing depth and base-level quality. For each sample, R1/R2 read counts, total read pairs, total raw reads, read length distribution, GC percentage, N percentage, Q20, and Q30 were calculated directly from the FASTQ files. Vendor quality-control reports were merged for effective-tag metrics, including combined reads, quality-filtered reads, non-chimeric effective tags, effective bases, effective-tag average length, GC, Q20, and Q30. Downstream 16S read assignment was summarized from the DADA2 ASV feature table and SILVA taxonomy. Human-genome mapping was not performed because this was targeted 16S rRNA V3-V4 amplicon sequencing rather than shotgun metagenomic sequencing.

Supplementary Figure S1

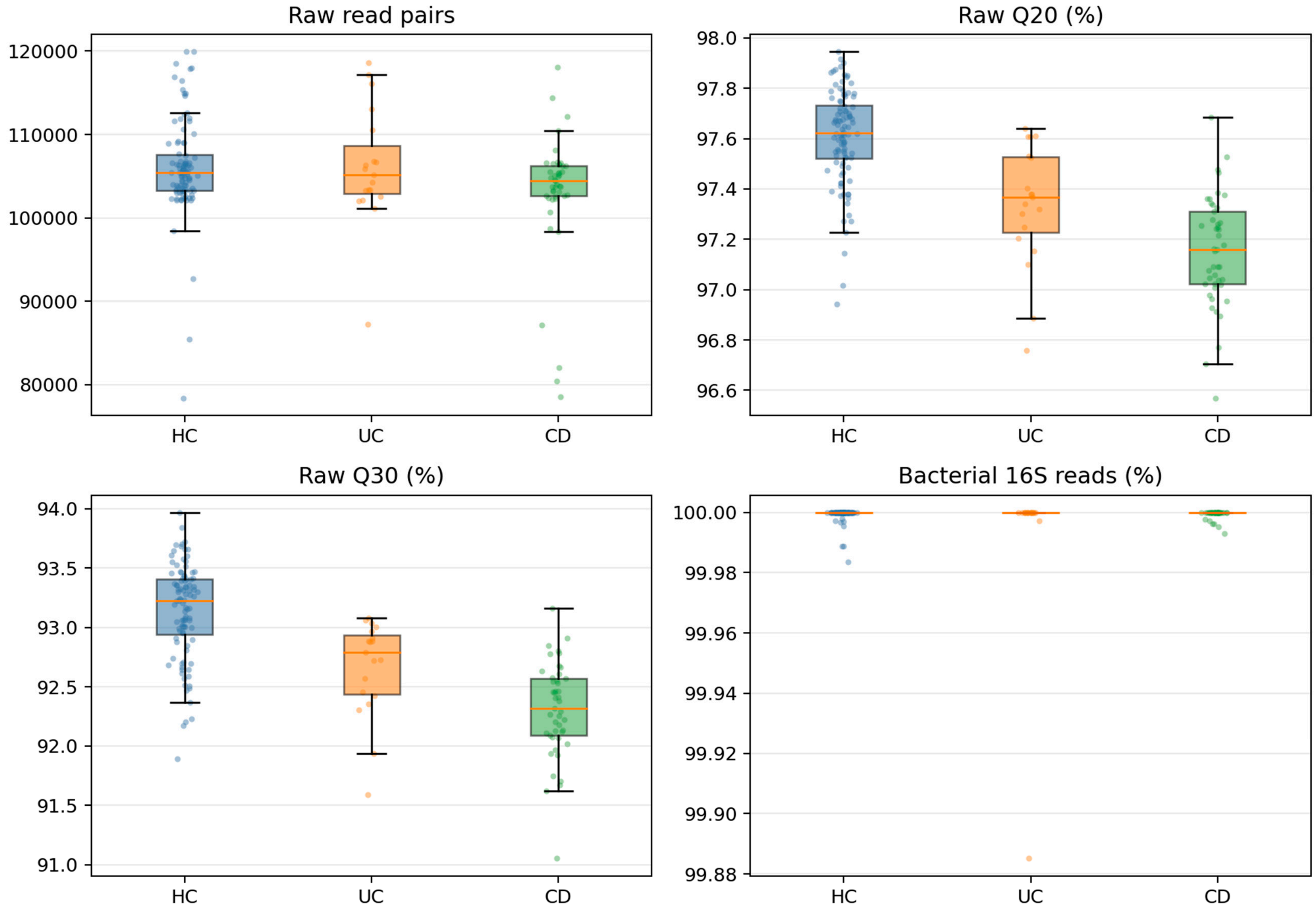

Supplementary Figure S1. FASTQ and 16S assignment overview across HC, UC, and CD groups. Panels show raw read pairs, raw Q20, raw Q30, and the percentage of ASV feature-table reads assigned to Bacteria.

Supplementary Table S1A. Group-level summary.

Group	n	Raw read pairs median	Raw read pairs range	Raw Q20 mean (%)	Raw Q30 mean (%)	ASV reads median	Bacterial reads median	Bacterial reads mean (%)	Effective tags median	AvgLen range (nt)
All	164	105,090	78,394-119,931	97.45	92.85	87,326	87,324	99.999	94,746	414.98-428.71
HC	100	105,396	78,394-119,931	97.61	93.14	91,984	91,984	99.999	97,064	418.79-428.71
UC	19	105,204	87,215-118,582	97.33	92.66	79,980	79,980	99.994	86,983	419.66-428.23
CD	45	104,444	78,520-118,105	97.15	92.31	74,196	74,196	99.999	83,628	414.98-427.39

Supplementary Table S1B. Field legend.

Field	Definition
sample_id	Sample identifier used in the manuscript metadata.
group	Manuscript analysis group: HC, UC, or CD.
raw_read_pairs	Number of paired-end read pairs in R1/R2 FASTQ files.
raw_total_reads_R1_plus_R2	Total raw reads across R1 and R2 FASTQ files.
raw_read_length_mode_bp	Modal read length for R1 and R2 in the delivered FASTQ files.
raw_q20_pct / raw_q30_pct	Percentage of FASTQ bases with Phred quality score >=20 or >=30.
raw_gc_pct / raw_n_pct	GC percentage and N-base percentage calculated from raw FASTQ sequences.
vendor_non_chimeric_effective_tags	Effective non-chimeric tags reported after merging, quality filtering, and chimera removal.
vendor_effective_avg_len_nt	Average length of effective merged tags in nt from vendor QC reports.
bacterial_16s_reads	DADA2/SILVA feature-table reads assigned to Bacteria.
archaea_16s_reads / unassigned_16s_reads	Feature-table reads assigned to Archaea or not assigned at kingdom level.
human_genome_mapped_reads	Not applicable for targeted 16S amplicon sequencing; no host-genome alignment was performed.

Supplementary Table S1C. Per-sample core sequencing and QC metrics.

Core per-sample metrics are shown below. The accompanying Excel file contains the complete field set, including raw file names, R1/R2 length min/max values, vendor combined/qualified reads, GC, Q20/Q30, and the explanatory human-mapping note.

Sample	Group	Raw pairs	R1+R2 reads	Raw Q20	Raw Q30	Effective tags	AvgLen	Bacterial reads	Bacterial %
BH001_1	UC	116,102	232,204	97.53	92.97	100,841	421.91	94,027	100.000
BH002_1	CD	110,449	220,898	97.09	92.07	90,914	424.02	78,589	100.000
BH003_1	CD	103,742	207,484	96.90	91.70	79,950	424.41	66,416	100.000
BH004	CD	104,033	208,066	96.57	91.05	86,451	427.39	73,738	100.000
BH005	CD	103,186	206,372	96.93	91.74	82,196	421.26	71,834	100.000
BH006	CD	102,340	204,680	97.01	91.97	77,474	422.40	65,298	100.000
BH007	UC	105,204	210,408	97.37	92.72	79,372	419.66	72,887	100.000
BH008	CD	103,694	207,388	97.34	92.58	83,493	423.25	71,138	100.000
BH009	UC	103,385	206,770	97.53	92.88	95,083	428.23	87,846	99.885
BH010	CD	106,425	212,850	97.25	92.38	86,249	421.89	77,244	100.000
DT01_1	HC	114,655	229,310	97.27	92.52	104,957	421.74	99,998	99.997
DT02_1	HC	102,158	204,316	97.23	92.47	93,663	424.78	86,076	100.000
DT03_1	HC	102,387	204,774	97.49	93.01	95,639	421.17	88,777	100.000
DT04_1	HC	106,295	212,590	97.67	93.23	95,596	419.74	91,577	100.000
DT05_1	HC	105,064	210,128	97.42	92.65	92,242	423.25	79,343	100.000
DT06_1	HC	103,705	207,410	97.70	93.30	92,675	421.61	87,673	100.000
DT07_1	HC	102,398	204,796	97.56	93.01	86,412	420.20	83,310	100.000
DT08_1	HC	110,050	220,100	97.66	93.26	95,413	418.79	90,316	100.000
DT09_1	HC	105,349	210,698	97.92	93.84	95,364	422.38	93,819	100.000
DT10_1	HC	103,907	207,814	97.72	93.33	88,031	425.17	81,871	100.000
DT11_1	HC	105,643	211,286	97.85	93.66	95,758	424.42	91,508	100.000
DT12_1	HC	105,389	210,778	97.84	93.60	98,111	424.20	93,302	100.000
DT13_1	HC	102,606	205,212	97.73	93.34	96,752	423.31	92,267	100.000
DT14_1	HC	107,701	215,402	97.56	92.99	98,189	421.69	93,333	100.000
DT15_1	HC	106,496	212,992	97.58	93.06	98,349	421.37	88,940	99.989
DT16_1	HC	102,423	204,846	97.70	93.31	94,725	425.41	89,674	100.000
DT17_1	HC	103,261	206,522	97.87	93.70	96,581	423.44	91,643	100.000

Sample	Group	Raw pairs	R1+R2 reads	Raw Q20	Raw Q30	Effective tags	AvgLen	Bacterial reads	Bacterial %
DT18_1	HC	103,057	206,114	97.41	92.61	94,768	424.96	88,160	100.000
DT19_1	HC	106,590	213,180	97.76	93.55	100,054	424.03	95,259	100.000
DT20_1	HC	103,931	207,862	97.67	93.36	96,417	428.71	91,526	100.000
DT21_1	HC	103,971	207,942	97.39	92.74	95,550	421.94	86,922	100.000
DT22_1	HC	108,980	217,960	97.64	93.31	100,427	423.89	95,807	100.000
DT23_1	HC	103,470	206,940	97.68	93.36	97,242	422.90	92,493	100.000
DT24_1	HC	119,911	239,822	97.71	93.41	106,646	420.64	101,016	100.000
DT25_1	HC	114,919	229,838	97.77	93.56	106,993	422.70	101,730	100.000
DT26_1	HC	106,236	212,472	97.50	93.00	94,937	422.03	90,729	100.000
DT27_1	HC	108,927	217,854	97.47	92.68	101,767	423.25	96,228	100.000
DT28_1	HC	104,976	209,952	97.75	93.46	98,500	424.90	92,660	100.000
DT29_1	HC	112,397	224,794	97.63	93.23	85,216	425.14	76,721	100.000
DT30_1	HC	103,871	207,742	97.59	93.17	84,206	421.23	74,667	100.000
DT31_1	HC	111,620	223,240	97.60	93.20	93,557	421.99	85,634	100.000
DT32_1	HC	116,471	232,942	97.37	92.70	88,499	426.03	77,708	100.000
DT33_1	HC	109,235	218,470	97.66	93.32	83,171	423.32	71,268	100.000
DT34_1	HC	103,016	206,032	97.95	93.97	85,916	424.20	77,661	100.000
DT35_1	HC	117,968	235,936	97.52	93.00	95,614	421.18	86,759	100.000
DT36_1	HC	105,402	210,804	97.60	93.25	84,923	423.07	80,632	100.000
DT37_1	HC	106,470	212,940	97.55	93.08	95,121	426.45	87,601	99.995
DT38_1	HC	106,079	212,158	97.69	93.41	88,630	424.22	80,876	100.000
DT39_1	HC	102,133	204,266	97.80	93.68	91,903	424.24	86,264	100.000
DT40_1	HC	105,040	210,080	97.68	93.43	94,115	423.46	87,047	100.000
DT41_1	HC	104,229	208,458	97.78	93.52	89,005	423.73	83,756	100.000
DT42_1	HC	103,461	206,922	97.85	93.72	94,498	423.71	89,308	100.000
DT43_1	HC	102,997	205,994	97.62	93.22	93,639	423.68	88,476	100.000
DT44_1	HC	105,637	211,274	97.54	93.09	95,375	422.74	89,085	100.000
DT45_1	HC	119,931	239,862	97.73	93.32	108,234	423.49	100,429	100.000
DT46_1	HC	116,881	233,762	97.66	93.37	96,440	425.56	85,039	100.000
DT47_1	HC	117,876	235,752	97.69	93.27	106,585	425.11	99,330	100.000
DT48_1	HC	106,481	212,962	97.65	93.34	95,446	421.39	89,412	100.000
DT49_1	HC	111,879	223,758	97.71	93.47	105,538	422.68	89,981	100.000
DT50_1	HC	105,116	210,232	97.62	93.30	93,425	423.68	89,239	100.000
NT01_1	HC	103,286	206,572	97.29	92.37	96,327	424.54	89,490	100.000
NT02_1	HC	102,258	204,516	97.53	93.05	91,242	423.22	84,457	100.000
NT03_1	HC	107,525	215,050	97.14	92.21	98,307	423.67	92,160	100.000
NT04_1	HC	104,735	209,470	97.34	92.64	94,831	422.80	88,611	100.000
NT05_1	HC	110,589	221,178	97.46	92.71	103,613	425.28	98,859	100.000
NT06_1	HC	104,511	209,022	97.62	93.06	99,546	423.99	93,543	100.000
NT07_1	HC	114,902	229,804	97.58	93.01	108,571	422.73	103,734	100.000
NT08_1	HC	104,826	209,652	97.65	93.08	98,865	424.02	94,181	100.000
NT09_1	HC	103,310	206,620	97.77	93.34	98,984	422.46	94,039	100.000
NT10_1	HC	106,795	213,590	97.67	93.24	99,589	419.81	95,854	100.000
NT11_1	HC	106,252	212,504	97.53	92.81	100,250	424.93	95,036	100.000
NT12_1	HC	104,566	209,132	97.89	93.70	98,797	422.09	95,897	100.000
NT13_1	HC	106,738	213,476	97.71	93.28	101,214	426.66	95,209	100.000
NT14_1	HC	102,103	204,206	97.51	92.88	95,909	423.46	90,097	100.000
NT15_1	HC	102,197	204,394	97.75	93.40	96,904	424.16	92,486	100.000
NT16_1	HC	105,895	211,790	97.60	93.06	101,455	423.09	95,745	100.000
NT17_1	HC	102,138	204,276	97.43	92.69	96,598	423.92	90,362	100.000
NT18_1	HC	103,536	207,072	97.82	93.46	97,088	424.53	92,496	100.000
NT19_1	HC	118,478	236,956	97.55	92.91	113,194	423.00	106,748	99.997
NT20_1	HC	105,966	211,932	97.55	92.97	101,164	423.82	94,919	100.000
NT21_1	HC	111,616	223,232	97.78	93.36	106,640	422.39	102,470	100.000
NT22_1	HC	102,279	204,558	97.86	93.61	97,715	426.47	91,951	100.000
NT23_1	HC	103,564	207,128	97.61	93.02	98,870	422.96	93,587	99.997
NT24_1	HC	103,929	207,858	97.38	92.57	99,139	421.86	92,814	100.000
NT25_1	HC	92,712	185,424	97.68	93.41	89,074	424.44	84,664	100.000
NT26_1	HC	106,629	213,258	97.49	93.16	94,978	418.97	91,453	99.984
NT27_1	HC	105,676	211,352	97.62	93.41	99,072	422.74	93,080	99.998
NT28_1	HC	115,365	230,730	97.42	92.96	106,555	422.22	101,576	100.000
NT29_1	HC	78,394	156,788	97.83	93.58	72,471	420.88	71,226	99.989
NT30_1	HC	107,258	214,516	97.78	93.47	102,490	425.91	96,842	100.000
NT31_1	HC	104,366	208,732	97.60	93.06	98,705	425.02	93,114	100.000
NT32_1	HC	105,745	211,490	97.70	93.21	100,133	424.04	93,405	100.000
NT33_1	HC	105,276	210,552	97.68	93.15	100,609	423.50	95,315	100.000
NT34_1	HC	108,985	217,970	97.79	93.44	101,610	424.16	97,944	100.000
NT35_1	HC	106,041	212,082	97.02	92.17	98,193	420.91	92,584	100.000
NT36_1	HC	109,038	218,076	96.94	91.89	100,809	423.83	93,910	100.000
NT37_1	HC	106,328	212,656	97.61	93.14	98,686	423.65	93,470	100.000

Sample	Group	Raw pairs	R1+R2 reads	Raw Q20	Raw Q30	Effective tags	AvgLen	Bacterial reads	Bacterial %
NT38_1	HC	103,297	206,594	97.46	92.67	96,233	424.67	90,690	100.000
NT39_1	HC	102,383	204,766	97.54	92.90	97,315	420.51	91,707	100.000
NT40_1	HC	85,495	170,990	97.38	92.49	79,275	421.67	72,257	100.000
NT41_1	HC	103,188	206,376	97.90	93.66	97,041	423.29	93,322	100.000
NT42_1	HC	112,011	224,022	97.36	92.51	104,352	424.96	99,672	100.000
NT43_1	HC	103,175	206,350	97.58	92.96	97,447	423.86	92,331	100.000
NT44_1	HC	102,715	205,430	97.27	92.23	96,525	421.91	90,919	100.000
NT45_1	HC	104,837	209,674	97.77	93.39	99,433	424.20	94,281	100.000
NT46_1	HC	106,309	212,618	97.38	92.59	100,448	421.08	92,016	100.000
NT47_1	HC	106,454	212,908	97.82	93.53	100,336	423.74	97,621	100.000
NT48_1	HC	112,620	225,240	97.52	92.85	106,047	424.36	99,184	100.000
NT49_1	HC	98,412	196,824	97.87	93.65	93,465	422.53	90,310	100.000
NT50_1	HC	105,433	210,866	97.79	93.45	100,305	424.10	95,072	100.000
TJ001	CD	102,716	205,432	96.95	92.01	90,759	421.04	85,144	100.000
TJ002	UC	106,315	212,630	97.34	92.79	86,536	424.24	78,736	100.000
TJ003	UC	118,582	237,164	97.10	92.36	96,371	423.73	86,948	100.000
TJ004	UC	105,923	211,846	97.25	92.57	84,584	422.26	76,557	100.000
TJ005	UC	110,583	221,166	97.38	92.88	93,714	424.02	86,898	100.000
TJ006	CD	102,582	205,164	97.06	92.20	83,628	423.30	78,271	100.000
TJ007	CD	105,052	210,104	97.09	92.32	76,511	420.38	68,711	100.000
TJ008	CD	105,531	211,062	97.02	92.12	80,095	423.32	69,156	100.000
TJ009	UC	102,532	205,064	97.32	92.73	79,611	423.50	71,667	99.997
TJ010	CD	106,613	213,226	97.27	92.66	79,577	422.92	69,831	100.000
TJ011	CD	103,076	206,152	97.04	92.18	80,673	419.72	74,196	100.000
TJ012	CD	114,421	228,842	96.96	92.07	88,883	422.54	77,794	100.000
TJ013	CD	105,338	210,676	97.26	92.41	85,338	421.24	76,723	100.000
TJ014	CD	106,284	212,568	97.04	92.14	83,772	424.03	70,820	100.000
TJ015	UC	103,358	206,716	97.40	92.88	86,983	424.17	79,980	100.000
TJ016	CD	98,737	197,474	97.08	92.27	82,013	425.56	69,435	100.000
TJ017	CD	108,157	216,314	97.15	92.41	91,497	423.10	83,542	100.000
TJ018	CD	106,626	213,252	97.02	92.11	88,423	421.63	83,475	99.998
TJ019	CD	100,645	201,290	97.36	92.78	85,441	425.17	78,809	100.000
TJ020	CD	102,671	205,342	96.70	91.62	88,727	425.91	73,732	100.000
TJ021	CD	80,419	160,838	97.24	92.55	65,497	422.85	58,428	100.000
TJ022	CD	105,548	211,096	96.98	92.09	82,271	421.35	73,002	99.997
TJ023	CD	78,520	157,040	97.09	92.29	66,793	426.00	57,350	100.000
TJ024	CD	103,864	207,728	96.77	91.67	77,606	424.17	63,603	100.000
TJ025	UC	87,215	174,430	96.76	91.59	67,651	425.25	56,801	100.000
TJ026	CD	106,696	213,392	96.91	91.92	82,809	423.58	74,396	100.000
TJ027	CD	87,189	174,378	97.25	92.63	72,310	421.52	64,530	100.000
TJ028	CD	104,444	208,888	97.16	92.46	87,696	423.38	77,300	99.996
TJ029	CD	98,328	196,656	97.16	92.47	80,175	422.38	68,844	100.000
TJ030	UC	101,168	202,336	97.15	92.42	84,061	422.93	77,058	100.000
TJ031	UC	106,767	213,534	96.89	91.94	93,299	421.33	80,278	100.000
TJ032	CD	82,094	164,188	97.22	92.61	67,693	421.86	62,372	99.995
TJ033	CD	102,414	204,828	97.36	92.85	86,226	422.58	78,404	100.000
TJ034	CD	103,722	207,444	97.02	92.13	83,511	421.50	81,659	100.000
TJ035	UC	104,300	208,600	97.38	92.91	85,905	424.33	78,625	100.000
TJ036	UC	103,255	206,510	97.64	93.06	78,548	426.44	70,686	100.000
TJ037	UC	113,078	226,156	97.61	93.03	92,864	423.43	86,283	100.000
TJ038	UC	106,676	213,352	97.61	93.01	93,216	425.05	85,423	100.000
TJ039	CD	102,180	204,360	97.68	93.16	87,937	424.12	81,913	100.000
TJ040	CD	106,542	213,084	97.28	92.54	86,809	421.36	78,207	99.996
TJ041	CD	106,231	212,462	97.38	92.57	92,392	423.89	85,114	99.993
TJ042	CD	103,306	206,612	97.34	92.46	90,102	422.18	80,699	100.000
TJ043	CD	105,017	210,034	97.48	92.80	90,871	420.94	84,674	100.000
TJ044	CD	102,631	205,262	97.18	92.22	83,282	424.36	70,477	100.000
TJ045	CD	104,508	209,016	97.31	92.45	80,257	421.59	71,116	100.000
TJ046	CD	118,105	236,210	97.32	92.53	95,613	422.95	83,130	100.000
TJ047	UC	102,134	204,268	97.30	92.46	82,767	425.67	73,489	100.000
TJ048	UC	117,144	234,288	97.61	93.08	101,295	422.97	97,010	100.000
TJ049	CD	106,436	212,872	97.24	92.26	89,906	423.41	78,113	100.000
TJ050	UC	102,057	204,114	97.21	92.31	90,976	423.06	86,185	100.000
TJ051	CD	105,140	210,280	97.38	92.68	82,531	414.98	68,095	100.000
TJ052	CD	104,840	209,680	97.05	91.94	79,933	422.17	69,717	100.000
TJ053	CD	105,390	210,780	97.46	92.79	96,644	424.77	80,492	100.000
TJ054	CD	112,143	224,286	97.53	92.91	103,267	421.43	96,887	100.000

Supplementary Table S1D. Raw sequencing-report samples excluded from the manuscript analysis.

Sample	RawPE	Combined	Qualified	Nochime	AvgLen	Q20	Q30	Reason
BH003-2	103,810	102,857	99,790	86,989	423.32	98.12	93.89	Not included in the final HC/UC/CD manuscript metadata.
BH002-2	105,521	104,567	101,818	80,717	424.35	97.95	93.47	Not included in the final HC/UC/CD manuscript metadata.
BH001-2	106,752	105,734	103,196	91,053	424.24	98.35	94.31	Not included in the final HC/UC/CD manuscript metadata.
